# Cannabinoid hyperemesis and pheochromocytoma hypertensive urgency: a case report 

**DOI:** 10.1186/s13256-024-04497-0

**Published:** 2024-03-19

**Authors:** Jeffrey M. Arendash, Cornel Chiu, Jocelyn Wang, Fred Mihm

**Affiliations:** Department of Anesthesiology, Stanford University, 300 Pasteur Dr., Stanford, CA 94305 USA

**Keywords:** Cannabinoid hyperemesis syndrome, Pheochromocytoma, Hypertensive urgency

## Abstract

**Background:**

This report presents a case of cannabinoid-induced hyperemesis syndrome causing repeated violent retching in a patient with a large (8 cm) adrenal pheochromocytoma resulting in hypertensive urgency.

**Case presentation:**

A 69-year-old white male patient with a previously diagnosed pheochromocytoma presented to the emergency department with nausea and vomiting and was found to have hypertensive urgency. Computed tomography scan did not show any acute abdominal pathology and history was inconsistent with a gastrointestinal etiology. Patient had a history of daily cannabinoid use for many years and repeated self-limited hyperemesis episodes, and thus a diagnosis of cannabinoid-induced hyperemesis syndrome was made. It was concluded that the likely explanation for the hypertensive urgency was from physical compression of his adrenal tumor during the episodes of retching resulting in a catecholamine surge. The patient was given antiemetics and admitted to the intensive care unit for blood pressure management. Blood pressure was initially controlled with phentolamine and a clevidipine infusion, then transitioned to oral doxazosin and phenoxybenzamine. Hyperemesis and abdominal pain resolved after 24 hours, and his blood pressure returned to baseline. The patient was discharged with the recommendation to stop all cannabis use. On follow-up, his blood pressure remained well controlled, and he subsequently underwent adrenalectomy for tumor removal.

**Conclusion:**

Hyperemesis can cause hypertensive events in patients with pheochromocytoma by increasing abdominal pressure, leading to catecholamine release.

## Introduction

Pheochromocytomas are rare neuroendocrine catecholamine-producing tumors that cause paroxysmal adrenergic episodes characterized by headaches, tachycardia, hypertension, and diaphoresis [1]. Hypertensive crisis is a dreaded, often fatal, complication of pheochromocytomas. Also known as pheochromocytoma multisystem crisis, these episodes are characterized by resistant hypertension with associated multiorgan failure and possible encephalopathy [2]. However, patients with pheochromocytomas can experience severe elevations in their blood pressure without end-organ damage. We report a case of hypertensive urgency from physical compression of a known pheochromocytoma secondary to violent retching due to presumed cannabinoid hyperemesis syndrome (CHS).

## Case Description

A 69-year-old white male patient with a past medical history of hypertension, coronary artery disease with prior stents, atrial fibrillation status post ablation, and a recent diagnosis of pheochromocytoma presented with hypertensive urgency in the setting of intense nausea and vomiting. A month and a half prior, pheochromocytoma was diagnosed during atrial fibrillation ablation, when he experienced periprocedural hypertensive emergency with systolic blood pressures > 250 mmHg, flash pulmonary edema, and hemoptysis. A computed tomography (CT) chest scan, ordered for hemoptysis, incidentally demonstrated an 8.5 × 7.5 × 7.2 cm left adrenal mass. Further history uncovered several years of intermittent pounding headaches, diaphoresis, nausea, and palpitations. Biochemical studies revealed total urine metanephrines elevated to 8193 mcg/24 hour (normal range 233–716) with elevations in both urinary metanephrines to 2909 mcg/24 hour (normal range 44–261) and normetanephrine to 5284 mcg/24 hour (normal range 138–521). Pulmonary edema and hypoxia resolved with diuresis. He was started on doxazosin 4 mg per os daily with close blood pressure monitoring, anticipating surgical adrenalectomy in approximately 6 weeks.

Our patient was asymptomatic after discharge until 2 days before scheduled surgery, when he developed worsening periumbilical pain, rated 4 out of 10 in severity, with nausea and vomiting. He reported ten episodes of emesis with repeated, severe retching over 12 hours prior to presentation to the emergency room. He denied headache, diarrhea, bloody stool, flank pain, hematuria, chest pain, shortness of breath, palpitations, diaphoresis, fever, chills, any recent changes to his diet, or sick contacts. His wife did not experience similar symptoms. He recalled similar emetic episodes over the last few years that resolved without medical intervention.

**Fig. 1:**
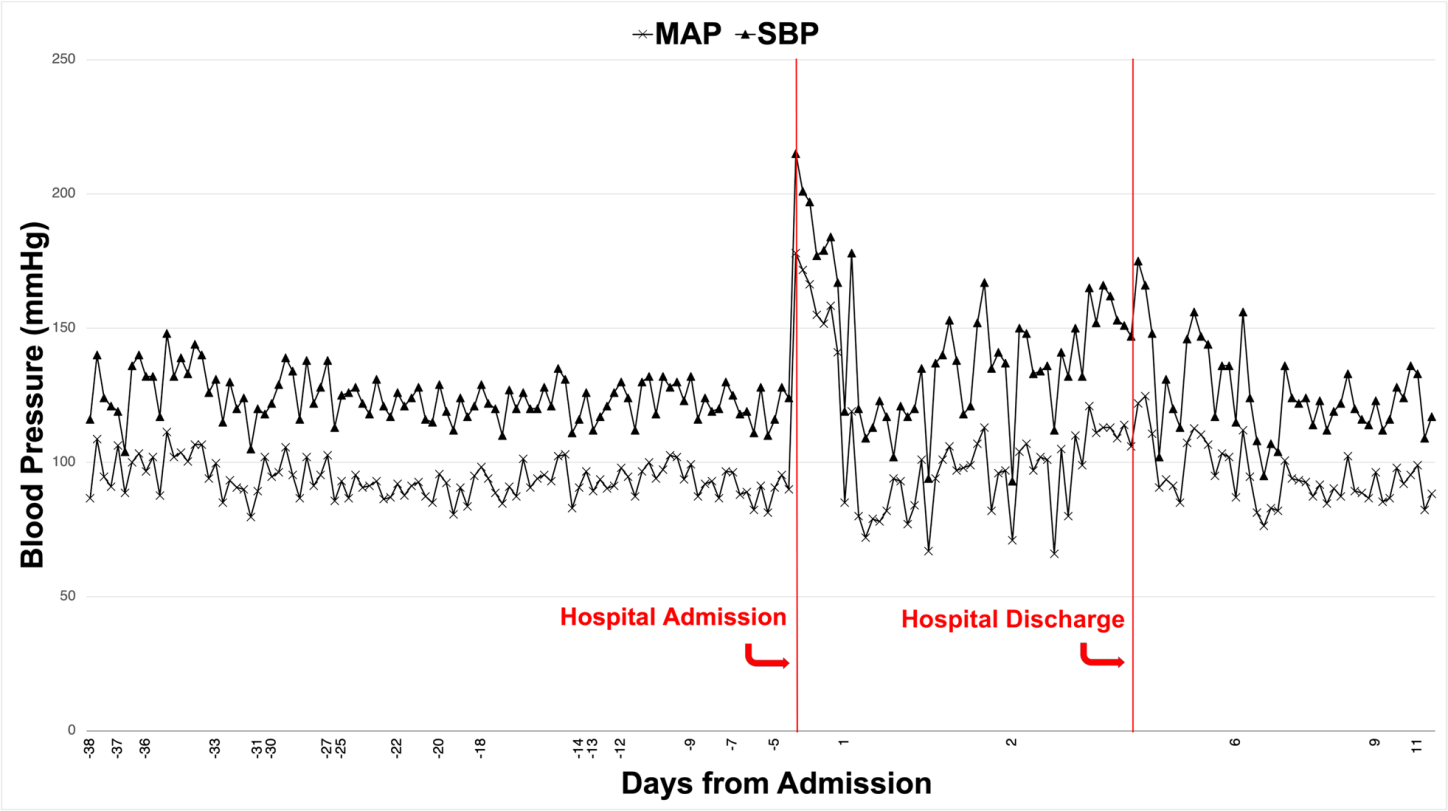
Blood pressure readings (*MAP* mean arterial pressure, *SBP* systolic blood pressure) prior to hospital admission, during hospital admission, and post-discharge. Increased blood pressure is noted on hospital admission compared with patient’s baseline, which normalized over the course of days upon resolution of retching

In the emergency department, his initial blood pressure was 201/113 mmHg with a heart rate in the 80s. His baseline blood pressure, with alpha adrenergic blockade, was a mean arterial pressure (MAP) between 80 mmHg and 100 mmHg (Fig. 1). He was afebrile with a room air oxygen saturation of 98% and an unremarkable physical exam. His abdomen was soft, nontender, nondistended, with normo-active bowel sounds in all four quadrants and negative for peritoneal signs, such as guarding or rebound tenderness.

Laboratory tests were unremarkable and without evidence of end-organ damage. His white blood cell count was 6.6 K/ul, with a normal hemoglobin, and mild thrombocytopenia at 119 K/ul. Electrolytes, creatinine, liver enzymes, lipase, lactate, and troponin I were all normal. Urinalysis showed a urine specific gravity of > 1.060 with 1 + ketones and protein, without blood, positive nitrites, or leukocyte esterase. Urine toxicology screen was positive for tetrahydrocannabinol (THC).

A CT abdomen and pelvis with intravenous contrast redemonstrated left adrenal mass measuring 7.1 × 8.7 × 7.1 cm, as well as diffuse diverticulosis without evidence of acute abnormalities.

The patient reported many years of frequent cannabis use. A year and half prior to presentation, he began using 87–88% THC with cannabidiol (CBD) in a smoke pen, taking 5–6 “hits” per day. Prior to that, he would smoke about 2–3 cannabis “joints” per day but denied ever eating cannabis. He also endorsed using chewing tobacco consistently over the last 30 years, using one can of tobacco over 4–6 days. He denied smoking tobacco, other recreational drugs, or alcohol.

**Fig. 2:**
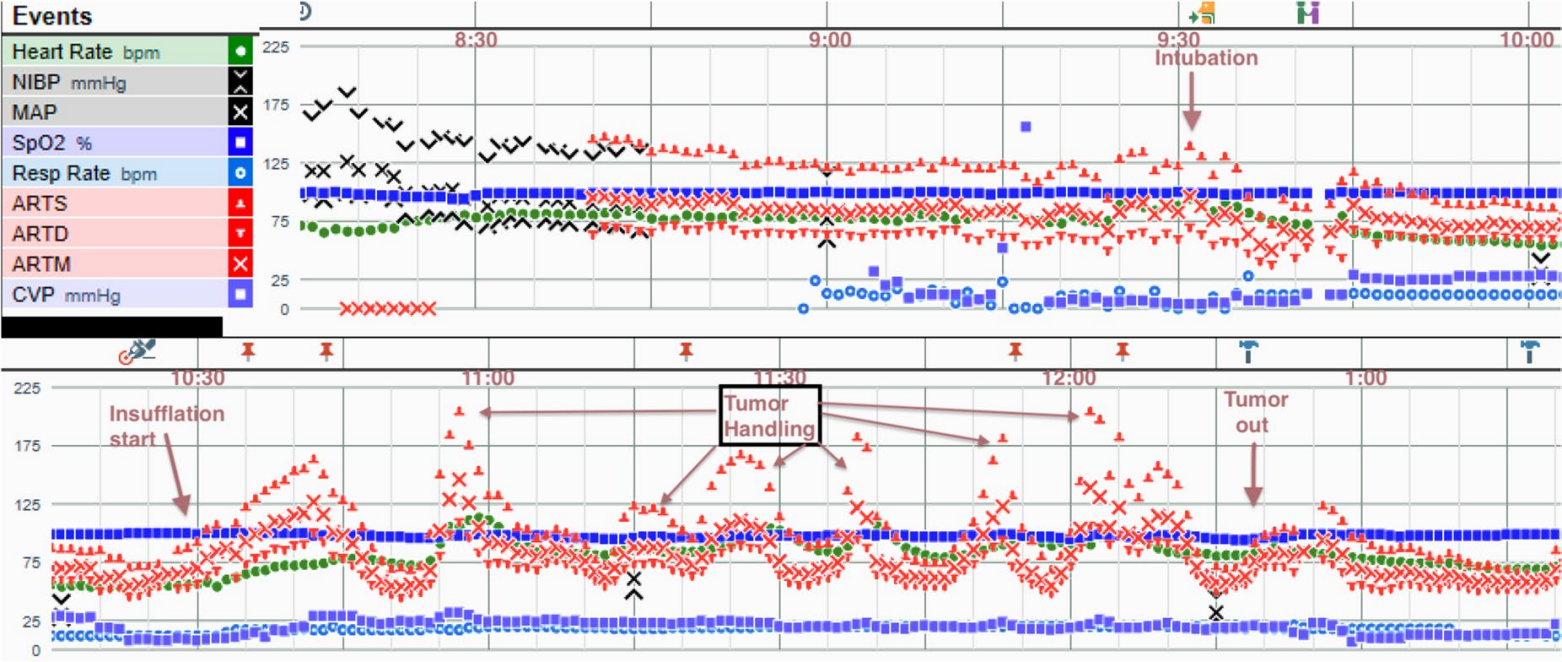
Intraoperative record during the patient’s adrenalectomy. Hypertensive episodes were observed with increased abdominal pressure from insufflation alone, then later during tumor handling, confirming that physical compression of the tumor from indirect pressure caused surges in blood pressure, even with the patient under a general anesthetic

Our patient had no evidence of new abdominal pathology or end-organ damage, ruling out pheochromocytoma multisystem crisis as a cause for his hypertension or gastrointestinal symptoms. Given that his blood pressure was well controlled prior to this episode, it was hypothesized that his hypertension was likely secondary to physical abdominal compression of his large adrenal tumor from forceful retching, leading to a release of catecholamines. Later, during surgical adrenalectomy under general anesthesia, a hypertensive event coincided with gas insufflation of the abdomen to 15 mmHg, an indirect affirmation of this hypothesis (Fig. 2). His large tumor also made it more likely that elevated abdominal pressure during retching could release catecholamines. Our initial differential diagnosis included ischemic bowel, cholecystitis, gastroenteritis, and CHS, but lab values and abdominal CT scan did not reveal any acute intraabdominal process that could explain his severe nausea and emesis. It should be noted that no evidence of intestinal ischemia was found that might have been a cause of nausea and vomiting. As the patient denied any sick contacts and his wife remained asymptomatic despite consuming the same food items, the diagnosis of viral or bacterial gastroenteritis became less likely. Noncompliance leading to poor blood pressure control was also considered, however, the patient was diligent in reporting blood pressures measured at home and confirming medication doses with regular emails to one of the authors. Upon eliciting the patient’s long history of recurring vomiting episodes and long-standing, frequent cannabis use, the diagnosis of CHS was the most likely explanation.

The patient was given intravenous phentolamine 5 mg, intravenous lorazepam 0.5 mg, and a lactated Ringer’s fluid bolus with improvement of his nausea. He was subsequently admitted to the medical intensive care unit (MICU) for close blood pressure monitoring and titration.

In the MICU, he required three doses of intravenous ondansetron 4 mg and one dose of intravenous lorazepam 0.25 mg for further nausea management. He was started on a clevidipine infusion, in addition to his home dose of per os doxazosin 4 mg daily for a MAP goal of less than 100 mmHg. Given that the patient’s MAP remained slightly elevated despite clevidipine and doxazosin, he was started on per os phenoxybenzamine 10 mg daily, which he tolerated well. By hospital day 2, the patient’s retching and vomiting stopped, and his diet was advanced. He was eventually weaned off clevidipine with per os doxazosin 6 mg/day and per os phenoxybenzamine 20 mg/day and discharged with the recommendation to stop cannabis and tobacco use.

Follow-up 1 week after discharge showed normalization of his blood pressure with no recurrence of his gastrointestinal symptoms (Fig. 1). He underwent successful adrenalectomy 2 weeks after discharge.

## Discussion

We present a patient previously diagnosed with a pheochromocytoma who experienced severe hypertensive episodes in the setting of protracted nausea, violent retching, and vomiting. CHS is a relatively underrecognized cyclic vomiting syndrome that occurs in chronic cannabinoid users. Generally, patients are male and use cannabis at least weekly and for greater than 1 year, particularly in those who have daily cannabis use [3]. They typically experience recurrent severe nausea and vomiting episodes and abdominal pain. The syndrome is characterized by three phases. The initial phase consists of nausea, fear of vomiting, abdominal discomfort, and anorexia. This is followed by the hyperemetic phase involving frequent, violent emesis and diffuse abdominal pain. This phase typically lasts about 24 hours until the recovery phase, when the symptoms gradually resolve [4]. The only proven treatment for CHS is cessation of cannabinoid use.

The exact pathophysiology of CHS is unknown. However, one theory is dysregulation of the endocannabinoid system, possibly from direct interaction with cannabinoid (CB-1) receptors in the gastrointestinal tract causing changes in motility. It is also postulated that sympathetic activation causes release of stored cannabinoids in fat, further perpetuating hyperemesis [5]. As such, patients with a pheochromocytoma may be at a greater risk for protracted hyperemetic sensitivity to cannabinoids given recurrent catecholamine surges.

Our patient had years of recurrent episodes of nausea and vomiting in the setting of chronic daily cannabis use that resolved without medical attention. The cyclic nature of his symptoms with clear symptom-free intervals correspond to the typical findings in CHS. The presence of a pheochromocytoma complicates this picture as pheochromocytoma itself can cause recurrent episodes of nausea and vomiting. The “clinical trap” for treating emesis in pheochromocytoma patients is that a routine antiemetic, metoclopramide (Reglan®), can precipitate life-threatening hypertensive crisis and is thus contraindicated in these patients [6]. During our patient’s episode, however, he did not experience headache or diaphoresis that are typical of the cyclic catecholamine surges related to pheochromocytomas, and abdominal CT imaging ruled out intestinal ischemia. The nausea and vomiting were sudden in onset, without prodrome. Additionally, the patient’s significantly elevated blood pressure resolved upon cessation of emesis, suggesting that the retching itself was causing the catecholamine surge rather than the reverse. A study by Iqbal *et al*. showed that intragastric and intravesicular pressures can increase to upwards of 280–290 mmHg during episodes of retching and vomiting, respectfully [7]. Generation of such high intraabdominal pressures can cause compression of secreting pheochromocytomas leading to catecholamine release and subsequent hypertension. After the CHS resolved, we postulate that additional alpha blockade was needed in the absence of the sedating effects of daily THC use.

## Conclusion

We propose that CHS was the likely etiology of this patient’s violent retching episodes leading to hypertensive urgency from physical compression of his pheochromocytoma, precipitating a catecholamine surge. We report a rare event in a rare disease and recommend heightened clinical awareness of other cases given the legalization of recreational cannabis use.

## Data Availability

All data generated or analyzed during this study are included in this published article.

## Abbreviations

CBD: Cannabidiol
CHS: Cannabinoid hyperemesis syndrome
CT: Computed tomography
IV: Intravenous
MAP: Mean arterial pressure
PO: Per os
